# Supplementary material for: Synthesis of Turbostratic Graphene with Micron-Sized Domains from Activated Charcoal by Fast Joule Heating
Source: Nanomaterials (Basel). 2025 Dec 15;15(24):1885. doi: 10.3390/nano15241885 (PMC12735881; doi:10.3390/nano15241885)
Supplement: Supplementary file 1 [file nanomaterials-15-01885-s001.zip › nanomaterials-4041984-supplementary.pdf]

Supplementary Information

Synthesis of Turbostratic Graphene with Micron-Sized Domains from Activated Charcoal by fast Joule Heating

Prokopiev Aisen Ruslanovich[a], Loskin Nikolay Nikolaevich[a], Vinokurov Pavel Vasilievich[a]

[a]Laboratory “Design-Center of Electronics «Sever»”, North-Eastern Federal University, Yakutsk, 677000, Russia*

Corresponding author, E-mail: aisenprokopiev@mail.ru

Supplementary Information Structure

1. Experimental Setup and Methods

Supplementary Figure S1. Photograph of the custom fast Joule heating (fJH) setup used for turbostratic graphene synthesis.

Supplementary Table S1. List of components and specifications for the fJH system.

2. Morphological Characterization

Supplementary Figure S2. SEM image of turbostratic graphene after fJH treatment, showing flake formation.

Supplementary Figure S3. AFM-profiles of turbostratic graphene.

3. Spectroscopic Analysis

Supplementary Figure S4. Raman spectra comparison: initial activated charcoal vs. turbostratic graphene.

Supplementary Figure S5. Raman spectra 2D peak.

Supplementary Figure S6. Raman mapping showing spatial distribution of D-band intensity across graphene domains.

Supplementary Figure S7. Raman mapping of G-band intensity distribution.

Supplementary Figure S8. Raman mapping of 2D-band intensity distribution.

4. Structural and Electrical Properties

Supplementary Table S2. Structural and electrical characterization of synthesized materials.

1.Experimental Setup and Methods

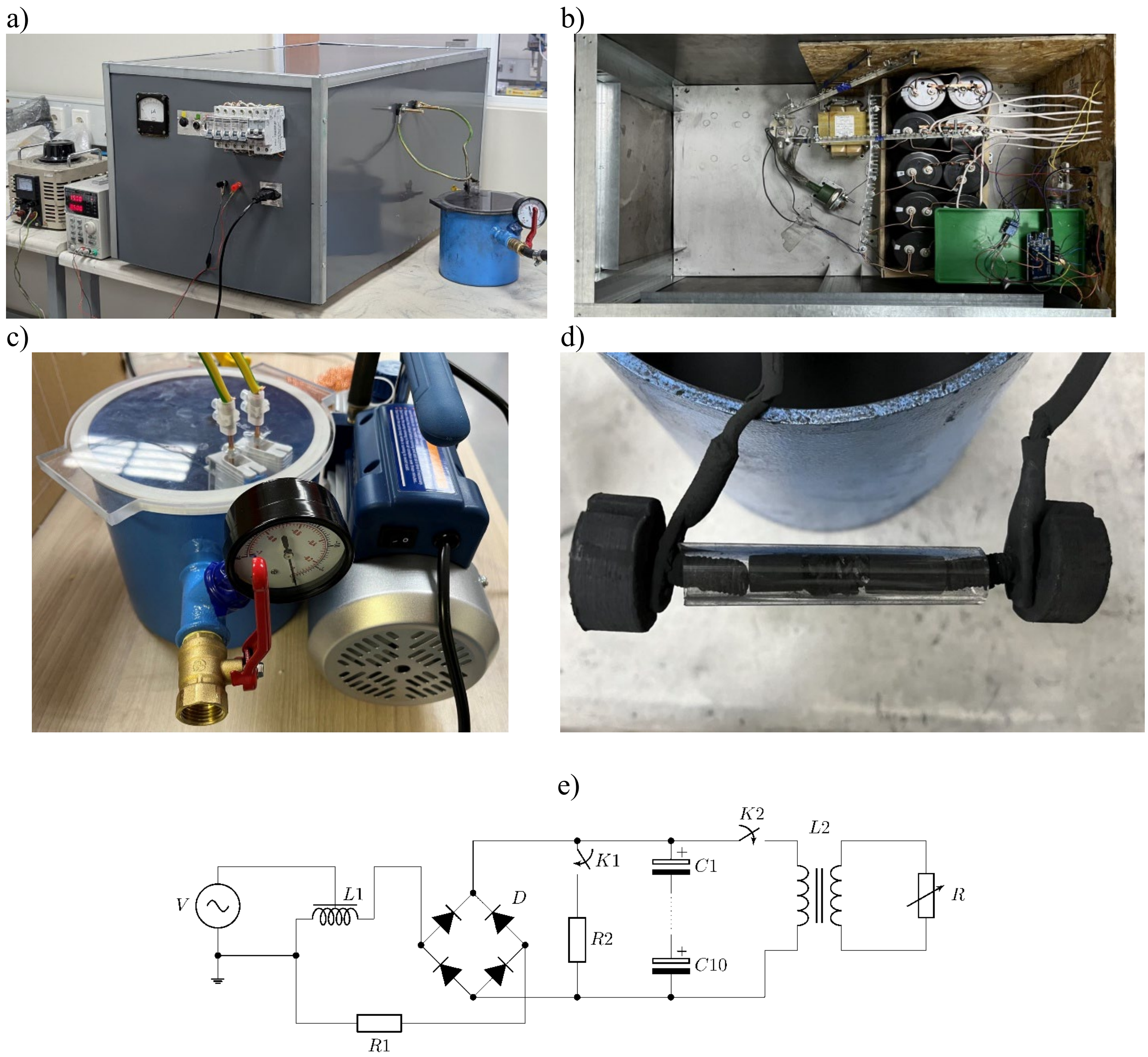

Supplementary Figure S1. Photograph of the custom fast Joule heating (fJH) setup used for turbostratic graphene synthesis: a) exterior view of the fJH setup; b) the inside of the fJH equipment; c) vacuum chamber with pump; d) quartz tube with electrodes; e) electrical schematic of the fJH setup.

Table S1. List of components and specifications for the fJH system.

Component	Model/Spec	Quantity
Capacitor bank	JIANGHAI CD135, 350 V, 180 mF	10
Inductor	Triad Magnetics C-80U, 24 mH	1
Relay	LEV200A5ANA, 900 V, 500 A	1
Rectifier diode	MDO500-12N1	1

Field effect transistor	N60G N-MOSFET, 600 V, 1 A	1
Quartz tube	“Eltemiks Co.” custom quartz tube, inner diameter 10 mm	1
Graphite electrodes	Custom electrodes, inner diameter 8 mm	2
Kill switch breaker	GENERICA BA47-29, 400 V	5
Charging system	TDGC2-3KVA, 0 – 250 V, 12 A	1
Power supply	Mastech HY3005C, 30 V, 3 A	1
Control panel	Arduino UNO with relay and voltage divider	1
Vacuum pump	Value VE-115N	1
Vacuum chamber	Cylindrical metal housing with epoxy resin coating with two taps on the end flanges. Capacity – 3 liters	1

2. Morphological Characterization

a)

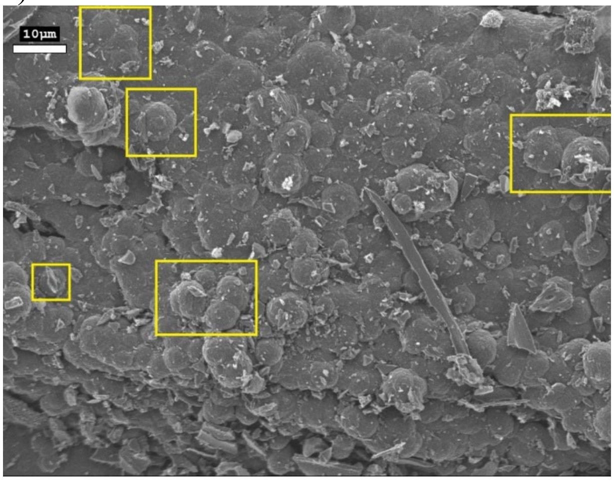

b)

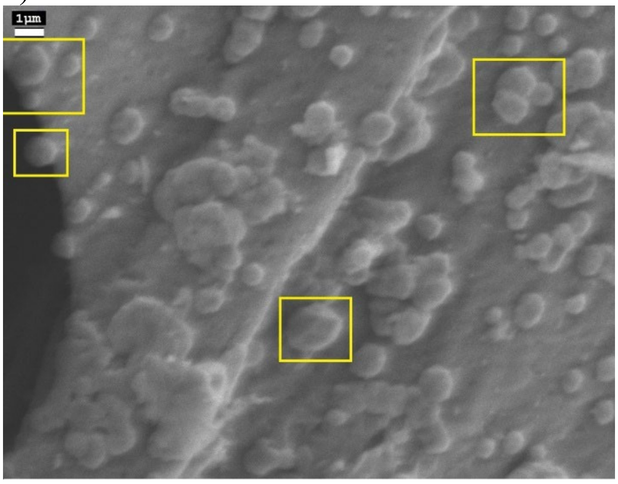

c)

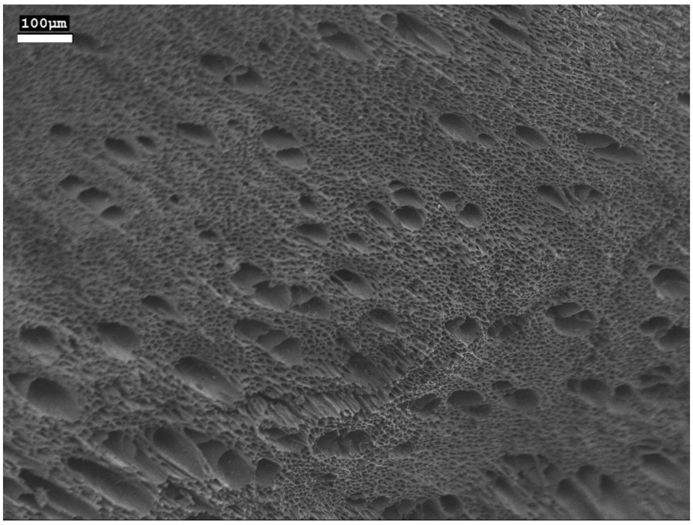

Supplementary Figure S2. SEM image of turbostratic graphene after fJH treatment, showing flake formation (SF 2a). The inserts to the Raman spectra figures (Fig. 5 in the article) in natural color (non-negative) are highlighted in yellow (SF 2b). Initial activated coal (SF 2c), showing porous surface.

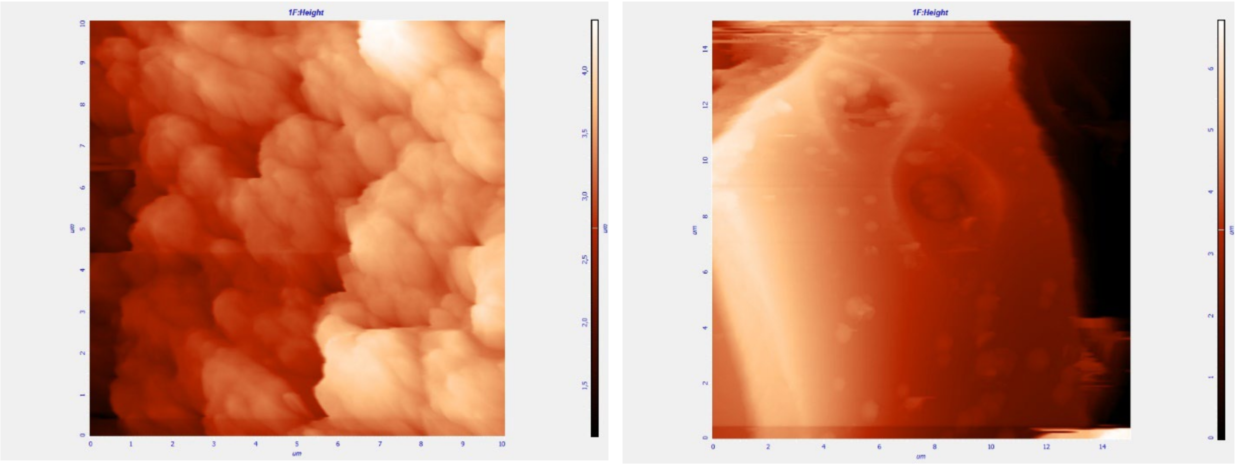

Supplementary Figure S3. AFM-profiles of turbostratic graphene.

3. Spectroscopic Analysis and XRD-spectra.

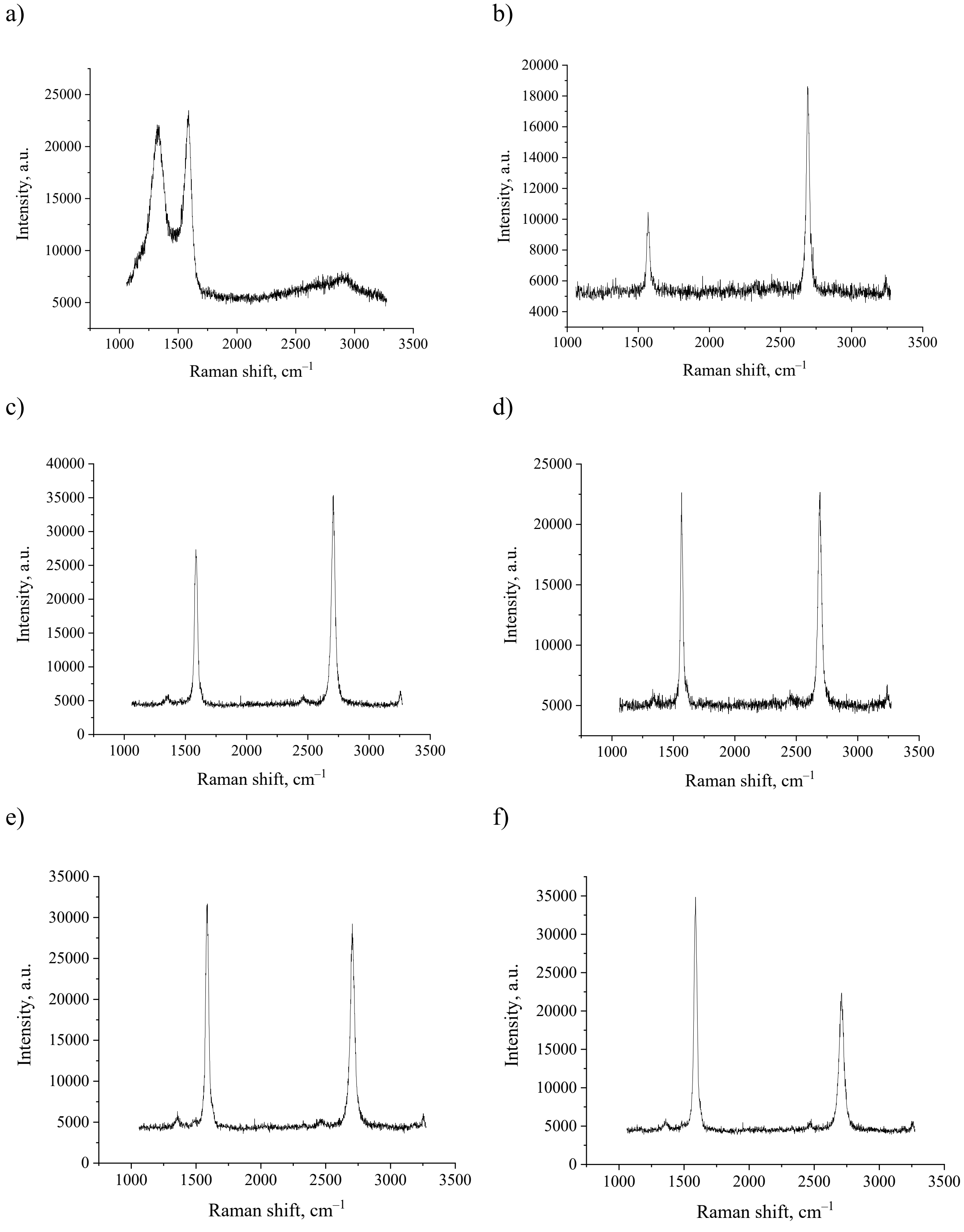

g)

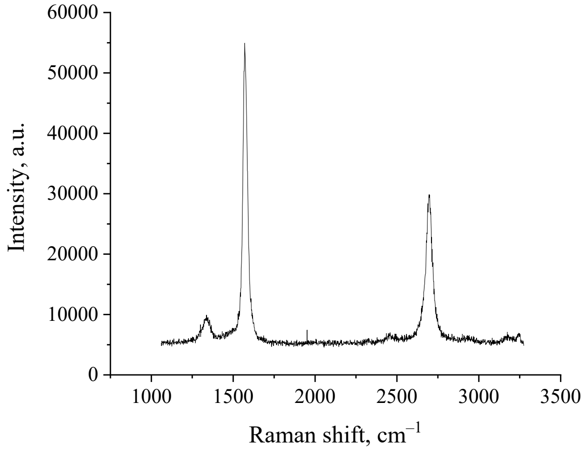

h)

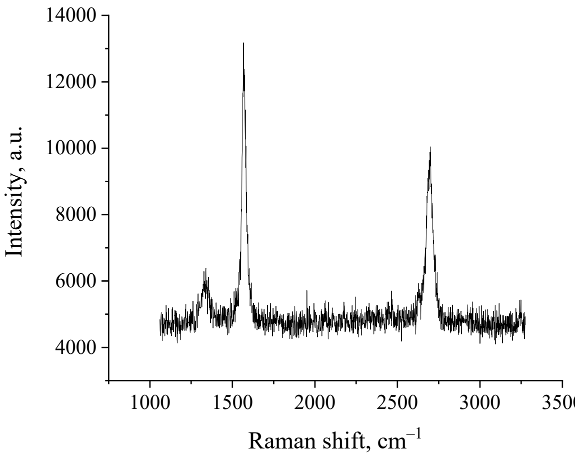

Supplementary Figure S4. Raman spectra comparison: (a) initial activated charcoal vs. turbostratic graphene in different structural ordering: (b,c) highly ordered domain, (d-e) medium ordered domain, (f-h) less ordered domain.

Supplementary Figure S4 presents the raw (unprocessed) Raman spectra (without background subtraction or smoothing) corresponding to the fitted data shown in Figure 5 of the main text.

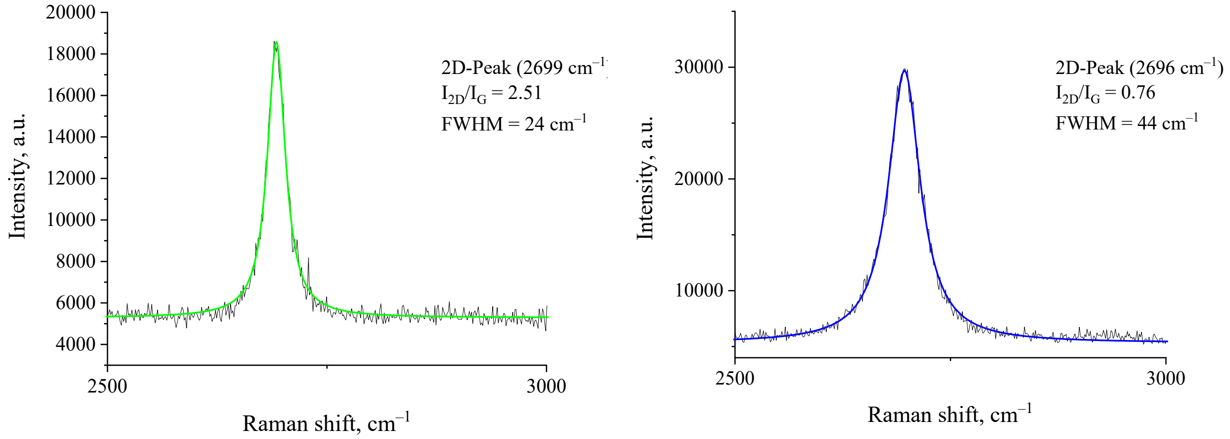

Supplementary Figure S5. Lorentzian fitting of the 2D Raman peak in turbostratic graphene domains with different crystallite sizes. Left: domain with large crystallite size (L_a>500 nm); Right: domain with small crystallite size (L_a<200 nm). The high coefficient of determination (R^2=0.99) confirms the adequacy of the single Lorentzian model for describing the 2D peak shape.

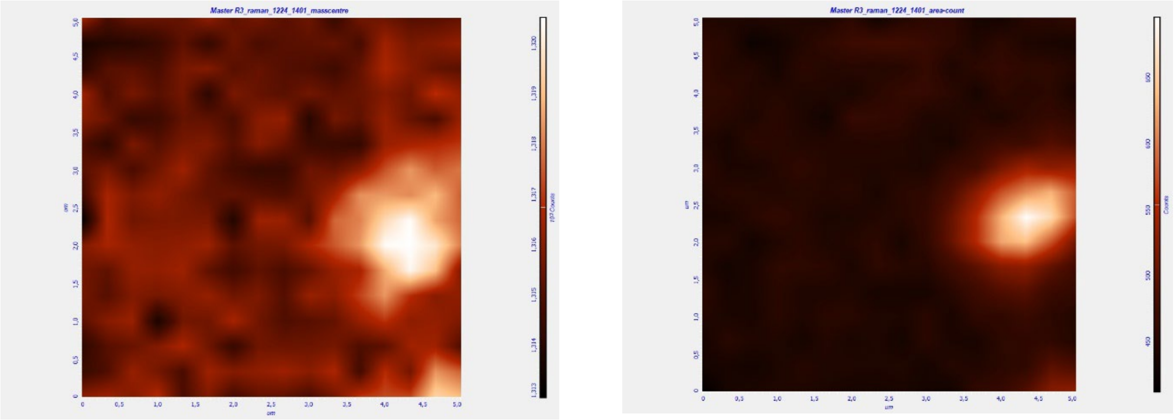

Supplementary Figure S6. Raman mapping showing spatial distribution of D-band intensity across graphene domains.

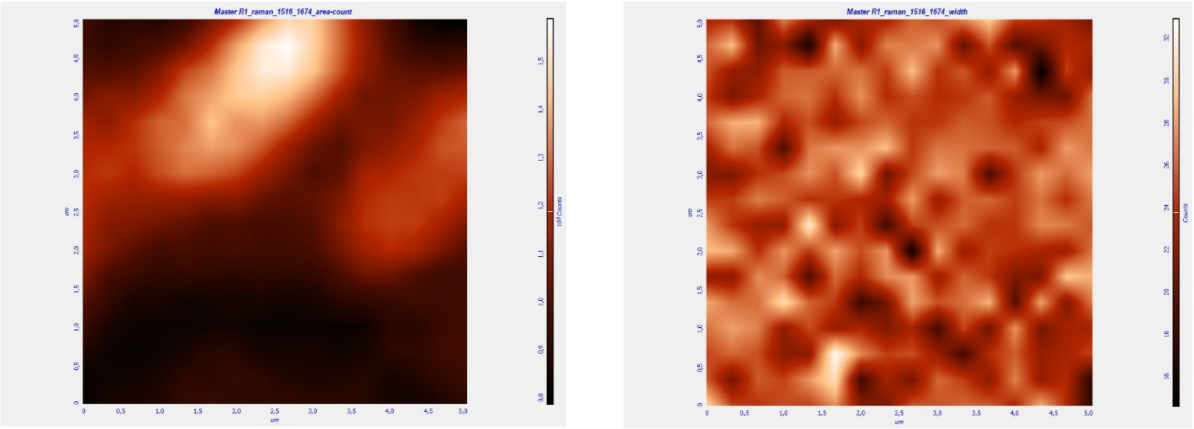

Supplementary Figure S7. Raman mapping of G-band intensity distribution.

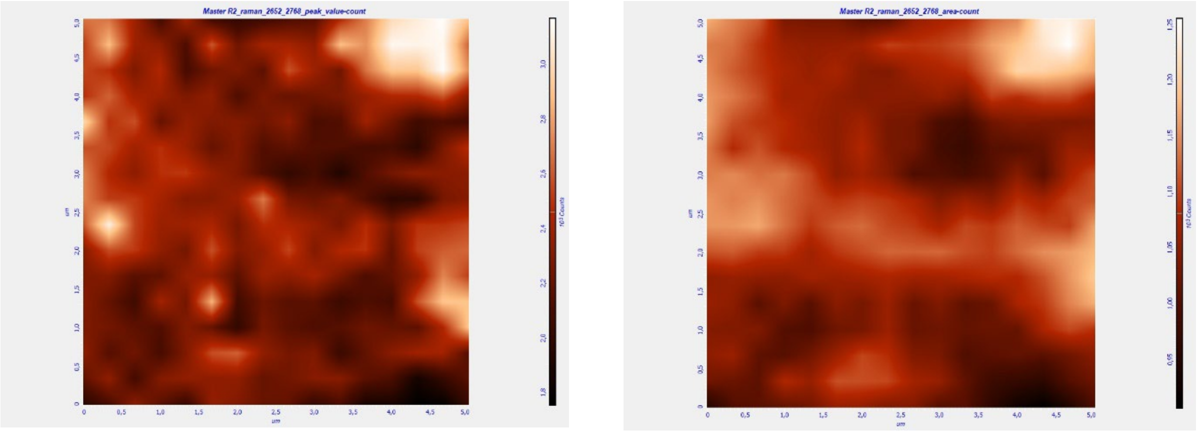

Supplementary Figure S8. Raman mapping of 2D-band intensity distribution.

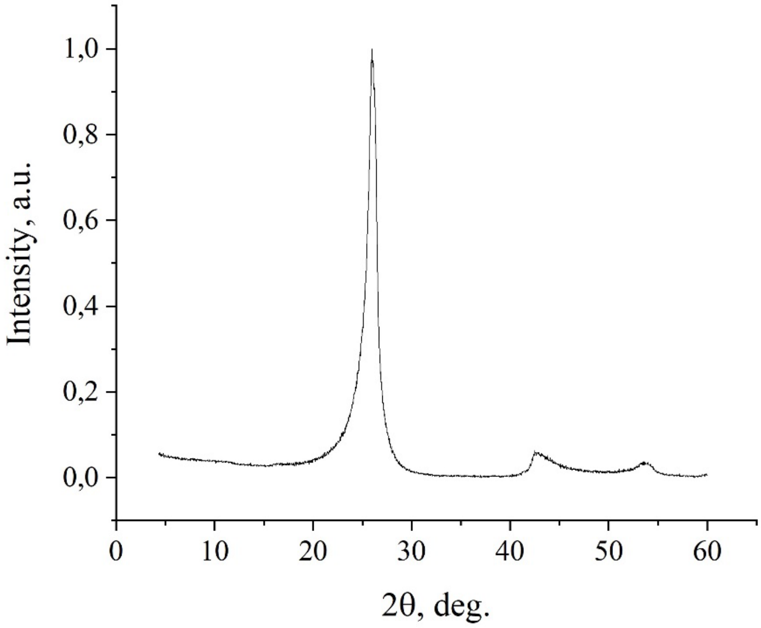

Supplementary Figure S9. Typical raw XRD-spectra activated charcoal after fJH.

4. Structural and Electrical Properties

Table S2. Structural and electrical characterization of synthesized materials

Sample Type	d_{002}, Å	Pellet resistivity (Ω·cm)	Pellet Density (g/cm³)	Measurement Condition
TG-1	3.430	0.50	~1.2	10 MPa, RT
TG-2	3.433	0.52	~1.2	10 MPa, RT
TG-3	3.432	0.51	~1.2	10 MPa, RT
…	…	…	…	…
TG-11	3.431	0.54	~1.2	10 MPa, RT
**TG Average**	**3.432 ± 0.002**	**0.51 ± 0.02**	**~1.2**	**10 MPa, RT**
**AC (Same)**	**-**	**2.52-5.13**	**~0.8**	**10 MPa, RT**

TG - turbostratic graphene; AC - activated charcoal; RT - room temperature

Bulk resistivity values of individual turbostratic graphene pellets synthesized from activated charcoal via fast Joule heating. All pellets were prepared under identical conditions (0.5 g powder, 10 MPa pressure, 18 mm diameter) and measured using the four-point probe method at room temperature. The average resistivity of 0.51 ± 0.02 Ω·cm represents a significant improvement over the initial activated charcoal (2.52-5.13 Ω·cm).
